# rBMP Represses Wnt Signaling and Influences Skeletal Progenitor Cell Fate Specification During Bone Repair

**DOI:** 10.1002/jbmr.29

**Published:** 2010-01-15

**Authors:** Steve Minear, Philipp Leucht, Samara Miller, Jill A Helms

**Affiliations:** 1 Department of Surgery, Division of Plastic and Reconstructive Surgery, Stanford School of MedicineStanford, CA, USA; 2 Department of Orthopedic Surgery, Stanford School of MedicineStanford, CA, USA

**Keywords:** bone morphogenetic proteins, Wnt, signaling, regeneration, repair, molecular

## Abstract

Bone morphogenetic proteins (BMPs) participate in multiple stages of the fetal skeletogenic program from promoting cell condensation to regulating chondrogenesis and bone formation through endochondral ossification. Here, we show that these pleiotropic functions are recapitulated when recombinant BMPs are used to augment skeletal tissue repair. In addition to their well-documented ability to stimulate chondrogenesis in a skeletal injury, we show that recombinant BMPs (rBMPs) simultaneously suppress the differentiation of skeletal progenitor cells in the endosteum and bone marrow cavity to an osteoblast lineage. Both the prochondrogenic and antiosteogenic effects are achieved because rBMP inhibits endogenous β-catenin-dependent Wnt signaling. In the injured periosteum, this repression of Wnt activity results in sox9 upregulation; consequently, cells in the injured periosteum adopt a chondrogenic fate. In the injured endosteum, rBMP also inhibits Wnt signaling, which results in the runx2 and collagen type I downregulation; consequently, cells in this region fail to differentiate into osteoblasts. In muscle surrounding the skeletal injury site, rBMP treatment induces Smad phosphorylation followed by exuberant cell proliferation, an increase in alkaline phosphatase activity, and chondrogenic differentiation. Thus different populations of adult skeletal progenitor cells interpret the same rBMP stimulus in unique ways, and these responses mirror the pleiotropic effects of BMPs during fetal skeletogenesis. These mechanistic insights may be particularly useful for optimizing the reparative potential of rBMPs while simultaneously minimizing their adverse outcomes. © 2010 American Society for Bone and Mineral Research.

## Introduction

In the last quarter century, substantial progress has been made toward understanding the molecular machinery governing fetal skeletal tissue development. Analyses of human and murine skeletal phenotypes have led to the identification of transcriptional regulators of chondrogenic and osteogenic cell fate commitment.(1–4) Careful scrutiny of the fetal growth plate has revealed novel interactions between growth factors, hormones, and matrix-remodeling enzymes that synchronize chondrocyte maturation with angiogenesis.(5–7) In addition, new regulators of skeletogenesis have been identified in large-scale genomic and microarray screens.(8–10) Collectively, these data have provided critical insights into the regulation of bone formation during embryonic development.(11)

An ongoing challenge in the field of skeletal tissue biology is to determine how these observations can be translated into therapeutic strategies to enhance adult skeletal tissue regeneration. Such strategies are often built on the basic principles underlying embryonic bone development because it has become increasingly evident that the mechanisms controlling fetal bone formation are similar to the mechanisms regulating adult bone repair and bone remodeling. The time scale and the distribution of bone-forming activity are different, but the cellular and molecular mechanisms governing chondrocyte, osteoblast, and osteoclast function are indistinguishable. Consequently, studies that investigate how undifferentiated pluripotent embryonic cells choose to proliferate versus adopt a chondrogenic or an osteogenic fate provides a “window” into that same decision that occurs as part of adult repair and remodeling.

Bone morphogenetic proteins (BMPs) were originally dentified as an “activity.” Urist discovered that soon after implantation into a muscle pouch, decalcified bone released a substance that induced host cells to differentiate into chondrocytes.(12,13) This osteoinductive effect is now attributable to the action of BMPs. The subsequent cloning and characterization of vertebrate BMPs revealed their pleiotropic functions. For example, BMPs are required for the earliest step in skeletal tissue formation, when cells undergo condensation.(14) BMPs also are required for multiple steps in the program of chondrogenesis by acting through Smads(15) to regulate *Msx* transcription factors and collagen gene expression.(16) BMPs also regulate fetal osteogenesis, in part, through their inhibitory effects on Wnt pathway activation.(17)

The striking ability of BMPs to induce endochondral ossification led to the development of recombinant proteins for the treatment of skeletal injuries and to augment autologous bone grafting. While some clinical studies indicate that recombinant BMPs stimulate bone healing,(18) there remains a significant disparity between the impressive results obtained in animal models and the less stellar effects observed in human trials.(19–21) In addition, their off-label use has led to a number of unanticipated and detrimental side effects.(22)

The basis for a disparity between human clinical data and preclinical trials in animals is unclear. Differences have been variously attributed to the method of recombinant BMP (rBMP) delivery, the number of responding cells at the implant site, and the extent of the skeletal injuries in animal models versus in humans. These explanations, however, cannot fully explain the discrepancies. The same delivery methods and delivery vehicles were used in preclinical trials and in animal models, but the effects on bone healing were dramatically different.(18,23–27) Furthermore, the efficacy of rBMPs was demonstrated in critical-size defects in animals that are comparable in severity to tibial nonunions in humans.(19,28)

Perhaps the most difficult issue to resolve is whether rBMPs have skeletal stem cell–specific effects. For example, in both human and mouse bone injuries, skeletal progenitor cells arise from multiple tissue compartments including the injured periosteum, endosteum, bone marrow cavity, vascular tissue, and surrounding musculature.(29–31) All these progenitor populations contribute cells to the healing skeletal injury, but whether they respond equivalently to rBMP is not known. We devised a skeletal injury model in which the contributions from these various tissue compartments could be readily distinguished from one another and then employed the same delivery method as is used in humans to treat the skeletal injuries with rBMP-2. Using transgenic mice and molecular and cellular analyses, we discovered that rBMP-2 represses endogenous β-catenin-dependent Wnt signaling. In the injured periosteum, repression of Wnt activity permits *sox9* and *collagen type II* upregulation followed by exuberant chondrogenesis. In the injured endosteum, however, repression of Wnt activity blocks *runx2* and *collagen type I* expression, leading to an arrest in osteoblast differentiation. In the surrounding musculature, rBMP induces phosphorylation of Smad 1/5/8 in muscle cells, which respond by proliferating and adopting a chondrogenic fate. These data from an adult injury site closely parallel the diverse functions of BMPs in fetal skeletal development and provide a framework for understanding the pleiotropic effects of rBMPs in bone repair.

## Methods and Materials

### Transgenic mice

We used the *Axin*^*lacZ/+*^ mouse (*Mus musculus*) as a reporter of Wnt responsiveness. In this mouse, the Wnt target *Axin2* was replaced with a copy of *LacZ* with an *Axin2* promoter.(32,33) Adult *Axin*^*lacZ/+*^ heterozygote mice were used in this study.

### Monocortical defect model

All procedures were approved by the Stanford Committee on Animal Research. These studies were conducted on mice between 2 and 5 months of age. We employed a monocortical tibial defect model to evaluate bone repair. After an appropriate level of anesthesia was reached, an incision was made over the anteroproximal tibia, and the tibial surface was exposed while simultaneously preserving the periosteal surface. A drill hole was created through a single tibial cortex with a high-speed dental engine (15,000 rpm) using a 1.0-mm drill bit (Drill Bit City, Chicago, IL, USA). The free edge of the cut muscle flap was replaced over the injury site with a single stitch, and the wound was closed surgically. Following surgery, mice received 0.1-mL subcutaneous injections of sterile saline and were allowed to ambulate freely.

### Sample preparation

Tibiae were harvested and skinned at the appropriate time points and decalcified in 19% EDTA until fully decalcified. Samples to be stained for Xgal activity then were soaked overnight in 30% sucrose and cryoembedded in 22-oxacalcitrol (OCT). These samples were sectioned at a thickness of 10 µm. All other samples were dehydrated in a graded ethanol series after decalcification. These samples then were soaked in xylene followed by paraffin. These paraffin-embedded samples then were sectioned at a thickness of 8 µm.

### Modified periosteal injury model

The monocortical defect was modified so that neither cortex was penetrated, leaving the bone marrow cavity uninjured. After an appropriate level of anesthesia was reached, an incision was made over the anteroproximal tibia, and the tibial surface was exposed while preserving the periosteal surface. Here, a 1-mm dremel drill bit was used with a drill engine to injure the periosteum of one cortex. The procedure resulted in the removal of an approximately 0.5 mm diameter piece of cortical bone; the bone marrow cavity was untouched.

### Delivery of rBMP-2

In treatment of the monocortical tibial defect, absorbable collagen hemostatic sponges (Integra LifeSciences Corporation, Plainsboro, NJ, USA) were cut to the dimensions of the injury site. Sponges were soaked in 1 µL of recombinant BMP-2 (Medtronics, Minneapolis, MN, USA) at a concentration of 1 mg/mL for 30 minutes at 4°C. After the drill injury was placed but before the muscle was apposed, the loaded sponge was inserted into the marrow space.

In treatment of the modified periosteal injury, the absorbable collagen hemostatic sponge was cut to twice the volume as specified earlier. This stabilized the sponge at the injury site and separated the muscle tissue from the periosteal tissue. The sponge was soaked in 2 µL of rBMP-2 and placed over the periosteal injury. The free edge of the muscle flap was secured over the sponge to the lateral tibial muscles as before, holding the sponge in place.

### Cellular analyses

*Immunohistochemistry*: The following description is a general procedure that we use for localization of protein within tissue sections; the precise protocol depends on the antibody being used. In general, tissue sections were dewaxed, followed by immersion in H_2_O_2_/PBS and washing in PBS. The sections were permeabilized with Ficin, followed by treatment with 0.1 M glycine. After further washing in PBS, the sections were blocked in ovalbumin or whole donkey IgG. Appropriate primary antibody was added and incubated overnight at 4°C and then washed in PBS. Samples were incubated with peroxidase-conjugated secondary antibody (Jackson Immunoresearch, West Grove, PA, USA) for 1 hour. DAB Kit (VectorLab, Burlingame, CA, USA) was used to develop color reaction. Here, we used antibodies to platelet endothelial cell adhesion molecule (PECAM) and proliferating cell nuclear antigen (PCNA).

*Evaluating β-galactosidase activity*: Tissue of interest was cryosectioned, fixed in 0.8% glutaraldehyde, and incubated with Xgal substrate overnight at 37°C.

*Quantifying Wnt responsiveness*: ×40 images of endosteum or periosteum adjacent to injury sites were taken. The injured cortical edge was used as a reference point to standardize the region of interest. Xgal^+^ cells were counted in the endosteal/periosteal ×40 field, and the data were evaluated as Xgal^+^ cells per area.

### In situ hybridization

All hybridization steps were done in RNase-free conditions. Tissue was embedded in paraffin and cut into 8.0-µm sections. Sections were dewaxed and washed in PBS. Sections then were treated with 0.2 N HCl and washed in PBS. Then sections were treated with proteinase K, washed in PBS, fixed in 4% paraformaldehyde, and washed again in PBS. Sections were treated with 0.5% acetic acid in 0.1 M trietholamine, washed, and dehydrated. The sections were incubated overnight at 52°C with the antisense RNA probe of interest in combination with in situ hybridization buffer (Ambion, Foster City, CA). All RNA probes were constructed antisense to the RNA sequence of interest and transcribed using digoxigen-labeled ribonucleotides and stored in hybridization buffer. Probes were used at approximately 1.0 µg/mL.

The sections were treated with stringency solutions of formamide, sodium citrate, and Tween-20 to remove unhybridized probe. The sections then were washed in maleic acid buffer (pH 7.5) and blocked with 2% blocking reagent (Roche, Indianapolis, IN, USA) and 10% lamb serum. Sections then were incubated with alkaline phosphatase–conjugated, anti-DIG Fab fragments in 2% blocking reagent and 1% lamb serum overnight at 4°C. Sections then were treated with tetramisole (Sigma, St. Louis, MO, USA) to block endogenous alkaline phosphatase and incubated in NTMT buffer with 10% polyvinyl alcohol (Sigma), 4-nitro blue tetrazolium (Roche), and 5-brom-4-chloro-3-indoyl-phosphate (Roche) to develop the color reaction.

### Histology and histomorphometry

Pentachrome and aniline blue were used to detect osseous tissues, as described previously.(34,35) Tibiae were collected on postoperative days 3, 6, and 14 to determine the volume of new bone in PBS and rBMP-2 samples. This was accomplished by generating paraffin sections, tissues were stained with aniline blue, and representative sections were analyzed as described below. In total, three to six tibiae were used for each condition.

The 1.0-mm circular monocortical defect was represented across approximately 120 tissue sections, each of which was 8 µm thick. Approximately 40 slides were generated from sections, and six to eight tissue sections were used for histomorphometric analysis. Each section was photographed using a Leica digital imaging system (×5 objective). The digital images were imported into Adobe Photoshop CS2 (Adobe Systems, Inc., San Jose, CA). The region of interest typically encompasses 10^6^ pixels. The number of aniline blue–stained pixels was determined using the Magic Wand tool (tolerance setting: 60; histogram pixel setting: cache level 1) by a single blinded investigator and confirmed by a second independent investigator. These data then were used to calculate the total volume of new bone in each callus. For quantifying the cartilage, safranin-O was used to stain proteoglycan-rich cartilage, and the preceding histomorphometry procedure was used with slight differences. Specifically, the tolerance was set to 40.

### Statistical analysis

A Student's *t* test was used to quantify differences described in this article. Error bars represent standard deviation. A number symbol (^#^) denotes a *p* value of less than .05, and an asterisk (^*^) denotes a *p* value of less than .01.

## Results

### rBMP-2 potentiates endochondral ossification in a skeletal injury

A skeletal defect model was used to investigate how rBMP-2 affected skeletal progenitor cells during the process of bone regeneration. This model was chosen because it effectively separates cellular contributions from the periosteum, the endosteum, and the surrounding muscle from the regenerative process.(34,36) Skeletal injuries were treated with rBMP-2-soaked sponges as specified by the manufacturer and as described in other animal models(26,37,38) and in humans.(24)

**Fig. 1:**
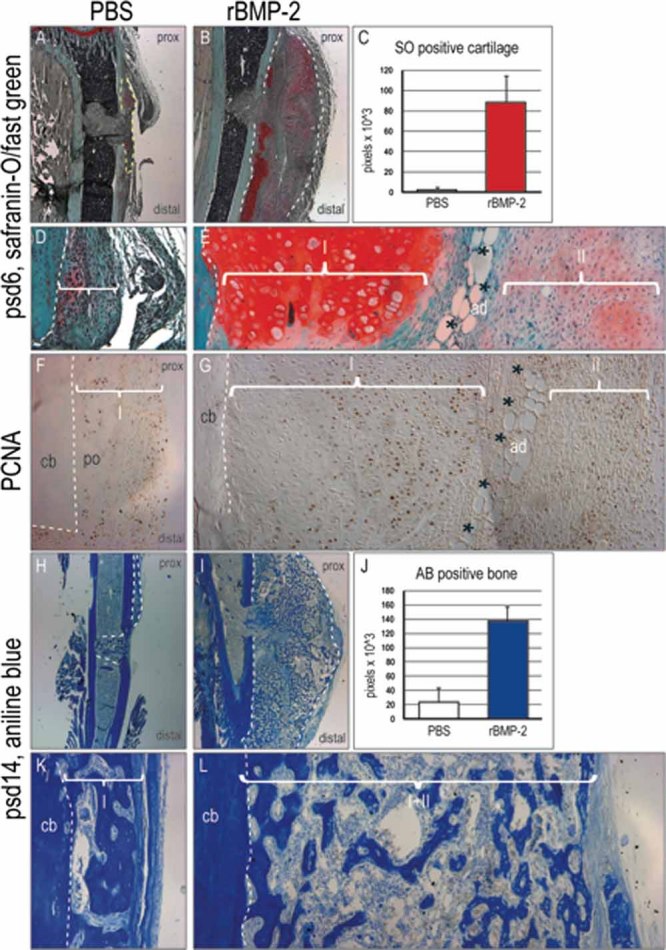
rBMP-2 induces a large extracortical callus via endochondral ossification. (*A*) safranin-O/fast green staining on postoperative day 6 cartilage in the PBS-treated injured periosteum signaling endochondral ossification (*dotted lines*). (*B*) rBMP-2 treatment induces a large cartilage callus superficial to the cortex. (*C*) Histomorphometric measurements show that compared with PBS, rBMP-2 induces a 31-fold increase in the size of the cartilage callus. (*D*) A ×40 view of safranin-O/fast green staining reveals minimal cartilage in the periosteal reaction (*po*) to injury in PBS-treated samples. (*E*) rBMP-2 treatment induces two large cartilaginous domains. One is the periosteal reaction (*I*) and the other is superficial (*II*) and is separated from one another by adipose/fibrous tissue (*asterisks*). (*F*) PCNA staining reveals proliferation in both the supraperiosteum and periosteum in PBS-treated controls. (*G*) rBMP-2 treatment reveals two domains of proliferating cells, a periosteal reaction and a second, supraperiosteal response. (*H*) Postoperative day 14 aniline blue staining of control samples shows robust osteogenesis in the bone marrow cavity (*dotted line*). (*I*) rBMP-2-treated samples exhibit a large bony callus located exclusively in the extracortical space; bone formation in the injury site is not detectable. (*J*) rBMP-2 treatment induces a 6-fold increase in the size of the bony callus. (*K*) In PBS-treated samples, aniline blue staining reveals the periosteal reaction on postoperative day 14. (*L*) By postoperative day 14, the two cartilage domains have coalesced into a single bony domain in the rBMP-2-treated samples (*white bracket*). ps = postsurgical; ad = adipogenic; cb = cortical bone; is = injury site.

On postoperative day 6, rBMP-2-treated samples exhibited a large cartilage callus, whereas PBS-treated samples showed only a small region of cartilage (*n* = 6 for each condition; Fig. 1*A,B*). Histomorphometric measurements demonstrated that rBMP-2 samples had a 31-fold increase in the cartilage callus volume compared with PBS controls (Fig. 1*C*). In the PBS-treated injury site, cartilage was evident only in the injured periosteum (*yellow dotted line*, Fig. 1*D*). In contrast, rBMP-2 samples exhibited two domains of cartilage: One domain extended from the cortical surface to a fibrous/adipogenic layer (i.e., domain I); the other cartilage domain extended from the fibrous/adipogenic layer to the subcutaneous tissue (domain II, Fig. 1*E*). In PBS controls, minimal PCNA immunostaining was detectable in the periosteum (i.e., domain I, Fig. 1*F*), whereas rBMP-2 samples showed robust PCNA immunostaining in domains I and II (Fig. 1*G*). Thus rBMP2 treatment induced cells in the periosteum and supraperiosteal space to proliferate and to adopt a chondrogenic fate.

On postoperative day 14, new bone occupied the PBS-treated bone marrow cavity, and the fibrocartilaginous tissue arising from the injured periosteum had been replaced by bone (*n* = 4; *dotted line*, Fig. 1*H*). rBMP-2-treated injury sites showed a different response: No new bone was present in the bone marrow cavity, and the entire extracortical region was encapsulated in a large bony callus (*n* = 3; *dotted line*, Fig. 1*I*). Histomorphometric measurements demonstrated that compared with PBS-treated controls, rBMP-2-treated samples had a 6-fold increase in the total amount of newly regenerated bone (Fig. 1*J*).

The location of the regenerate also provided clues as to the origins of the new bone. For example, in PBS-treated controls, new bone was found primarily in the bone marrow cavity (Fig. 1*H*), indicating its origin from osteoprogenitor cells in the endosteum.(36) A small amount of new bone was detectable in the periosteal region of the PBS-treated samples (Fig. 1*K*), which likely arises from osteoprogenitor cells in the periosteum.(39) In rBMP-2-treated samples, the bony regenerate was found exclusively in the extracortical region (Fig. 1*L*), indicating its derivation from the periosteum and from the surrounding soft tissues. No new bone (or cartilage) was detected in the bone marrow cavity. To understand the molecular basis for these disparate effects, we examined each domain in more detail, focusing first on the bone marrow cavity and then on the extracortical domains.

### rBMP-2 prevents intramembranous ossification in the bone marrow space

**Fig. 2:**
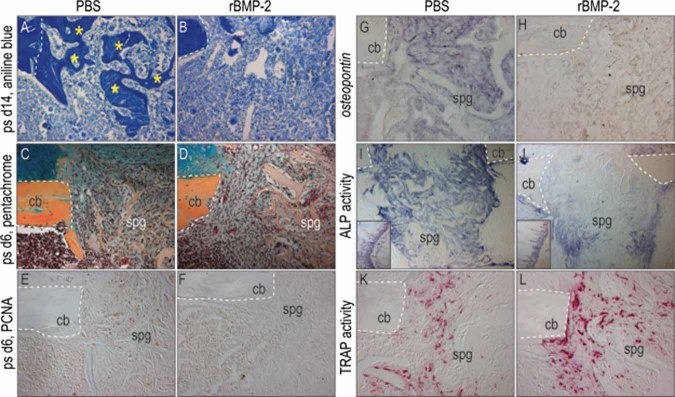
rBMP-2 does not induce bone formation in the marrow cavity. (*A*) In controls, new bone forms primarily in the bone marrow cavity, which begins to bridge the defect (*asterisks*), but (*B*) in rBMP-2-treated samples, there is no evidence of osteoid matrix in this location. (*C*, *D*) Pentachrome staining on postoperative day 6 reveals no obvious differences in the placement of the collagen sponge, the extent of cellular infiltrate, or the amount of vascularization between control and rBMP-2-treated samples. Despite their histologic equivalency, (*E*, *F*) the number of PCNA-immunopositive cells is increased in controls compared with rBMP-2-treated samples. In addition, (*G*, *H*) controls show higher levels of *osteopontin* expression than rBMP-2-treated samples. (*I*) PBS-treated controls also show more extensive alkaline phosphatase activity than (*J*) rBMP-2-treated samples. Insets in panels *I* and *J* illustrate equivalent levels of alkaline phosphatase staining in the growth plates of both tissue sections. In contrast, (*K*, *L*) TRAP activity is reduced in controls compared with rBMP-2-treated samples. Dotted line outlines cortical bone. ps = postsurgical; cb = cortical bone; spg = sponge.

Normally, after injury, cells in the bone marrow cavity proliferate and then differentiate into osteoblasts and deposit a mineralized matrix (Fig. 2*A*, *asterisks*). This response was not observed in rBMP-2-treated injuries (Fig. 2*B*). We evaluated each injury site thoroughly but did not observe bone matrix in the bone marrow cavity of rBMP-2-treated samples (*n* = 3). To understand the basis for this differential response in the bone marrow cavity, we examined injuries at earlier time points. On postoperative day 6, remnants of the collagen sponges still were evident both PBS- and rBMP-2-treated injury sites, but PBS-soaked sponges were filled with cells and their extracellular matrix (Fig. 2*C*). rBMP-2 soaked sponges showed little cellular infiltrate (Fig. 2*D*). Cells in the PBS-soaked sponges were proliferating (Fig. 2*E*), whereas no PCNA immunostaining was detectable in the rBMP-2-soaked sponges (Fig. 2*F*). These results are in striking contrast to the robust cell proliferation elicited by rBMP-2 in the injured periosteum (Fig. 1); here, cells in the bone marrow cavity showed almost no proliferation in response to the same rBMP-2 stimulus.

Normally, by postoperative day 6, cells in the bone marrow cavity have begun to differentiate into osteoblasts, which was illustrated by the broad domain of *osteopontin* expression in PBS-treated samples (Fig. 2*G*). *Osteopontin* expression was not detected in the bone marrow cavity of BMP-2-treated samples (Fig. 2*H*). PBS-treated samples also showed evidence of robust alkaline phosphatase activity in the bone marrow cavity (Fig. 2*I*). Alkaline phosphatase activity was lower in rBMP-2-treated samples (Fig. 2*J*). These two assays indicated that rBMP-2 treatment caused cells in the endosteum and bone marrow, which normally contribute to the bony regenerate, to arrest prior to their differentiation into osteoblasts. Consequently, these cells did not contribute to the bony regenerate, as they did in the PBS-treated samples. We also noticed that rBMP-2-treated samples had more robust TRACP activity than PBS samples (Fig. 2*K, L*), which is in keeping with reports of BMP-mediated activation of osteoclasts.(21,40) Taken together, these data demonstrated that rBMP-2 exposure blocked the osteogenic differentiation of cells from the endosteum and bone marrow. We next explored the molecular basis for this cellular response.

### rBMP-2 represses osteoblast differentiation in a site-specific manner

**Fig. 3:**
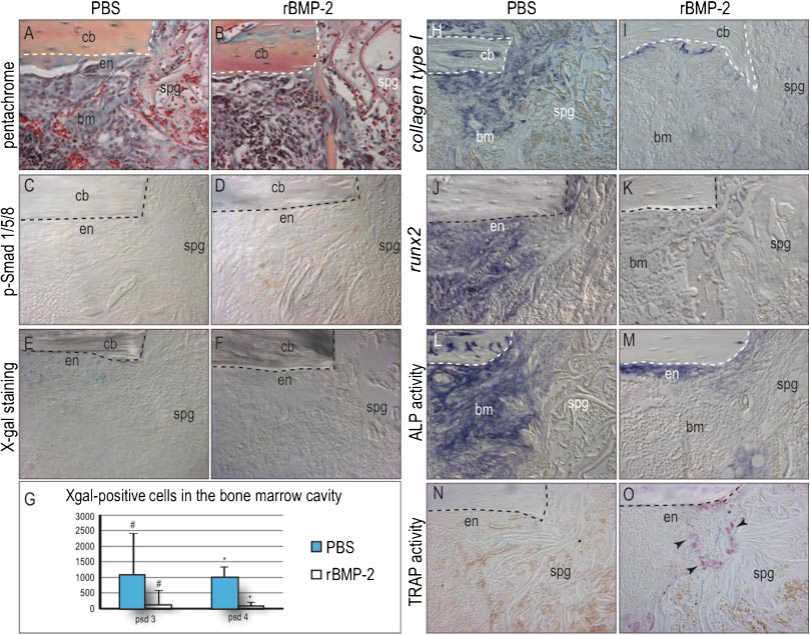
rBMP-2 suppresses differentiation of osteoprogenitors in the bone marrow cavity. (*A*, *B*) Pentachrome staining of the bone marrow cavity on postoperative day 3 shows evidence of the collagen sponge carrier as well as a dense cellular mass with abundant red blood cells. PBS-treated controls show more extracellular matrix than rBMP-2-treated samples. (*C*) Immunostaining for phospho-Smad 1/5/8 reveals no cells responding to a BMP-2 stimulus. (*D*) More cells, relative to controls, are responding to rBMP-2 in the endosteum of rBMP-2-treated samples on postoperative day 3. (*E*) *Axin2^lacZ/+^* mice were used to map β-catenin-dependent Wnt signaling in the bone marrow cavity. PBS-treated control endosteum demonstrates extensive Wnt responsiveness. (*F*) rBMP-2 abrogates the β-catenin-dependent Wnt responsiveness in the endosteum. (*G*) Quantification reveals a statistically significant reduction of Wnt responsiveness on postoperative days 3 and 4. (*H*, *J*) Control samples demonstrate expression of early markers of osteogenesis, including *collagen type I* and *runx2*, whereas (*I*, *K*) rBMP-2-treated samples exhibit lower levels of gene expression. (*L*) PBS-treated samples show robust alkaline phosphatase activity in the marrow space, but (*M*) rBMP-2 downregulates this activity. (*N*, *O*) rBMP-2-treated samples exhibit more TRAP activity than PBS-treated samples. Dotted line outlines cortical bone. cb = cortical bone; en = endosteum; spg = sponge; bm = bone marrow. ^#^*p* < .05; *p < .01.

PBS- and rBMP-2-treated samples were collected on postoperative day 3, a stage during the healing process that precedes overt osteogenesis (Fig. 3*A, B*). We made three discoveries. First, using phospho-Smad 1/5/8 immunostaining, we confirmed that cells in the injured bone marrow cavity responded to rBMP-2; in comparison, cells in the PBS-treated samples showed very little phospho-Smad immunoreactivity (Fig. 3*C, D*). Second, we determined that rBMP-2 treatment inhibited endogenous Wnt signaling. We first used an antibody to the phosphorylated form of β-catenin, which identifies the protein when it is targeted for degradation. The specificity of the antibody was demonstrated by the immunolabeling of chondrocytes in the growth plate (Supplemental Fig. 1*A*, *B*), but the antibody showed only nonspecific staining in the injured bone marrow (Supplemental Fig. 1*C*, *D*). We also tested an antibody to dephosphorylated β-catenin that identifies the protein in its active state. The specificity of the antibody was confirmed by positive immunostaining in growth plate osteoblasts (Supplemental Fig. 1*E*, *F*), but again, the injured bone marrow cavity showed only nonspecific staining (Supplemental Fig. 1*G*, *H*).

We then employed a genetic approach to determine if rBMP-2 treatment affected the endogenous Wnt pathway. We did this by generating skeletal injuries in *Axin2*^*LacZ/+*^ transgenic mice, in which the *LacZ* gene is under control of the Wnt target *Axin2*.(33) The *LacZ* gene product, which is detected by Xgal staining, therefore serves as a readout of β-catenin-dependent Wnt activation.(41,42) In PBS-treated samples, the endosteal region of the bone marrow cavity adjacent to the injury site was Xgal^+^ (Fig. 3*E*). In rBMP-2-treated samples, Xgal activity was minimal or undetectable (Fig 3*F*). Quantification of Xgal activity confirmed the significant decrease in Wnt responsiveness in the rBMP-2-treated samples (*p* < .05; Fig. 3*G*). This significant reduction in Xgal activity also was seen on postoperative day 4 (*p* < .01; Fig. 3*G*).

Wnt signaling is required for bone formation in the injury marrow cavity.(34) In PBS-treated samples, the distribution of Xgal^+^ cells coincided with the expression of *collagen type I* (Fig. 3*H*) and *runx2* (Fig. 3*J*), two markers of early osteoblast differentiation. In addition, PBS-treated samples showed evidence of robust alkaline phosphatase activity (Fig. 3*L*), an indicator of mineralization.(43) In rBMP-2-treated samples, there was no detectable expression of either *collagen type I* (Fig. 3*I*) or *runx2* (Fig. 3*K*) and only minimal alkaline phosphatase activity (Fig. 3*M*). As observed previously (Fig. 2*J*), rBMP-2-treated samples showed more robust TRACP activity than controls (Fig. 3*N* compared with Fig. 3*O*), but this increased osteoclastic activity was not associated with an osteogenic response in the bone marrow cavity (Fig. 2). The basis for this BMP-mediated effect on osteoclastogenesis was explained in part by changes in the expression of RANKL, a positive regulator of osteoclastogenesis, and osteoprotegrin (OPG), a negative regulator of the same program.(44) Relative to PBS-treated controls, the rBMP-2-treated samples showed an increase in RANKL immunostaining and a reduction in OPG immunostaining (Supplemental Fig. 2 *A–D*). Thus rBMP-2 treatment represses endogenous Wnt signaling in the bone marrow cavity, leading to repression of osteoblast differentiation and block of bone regeneration in this locale.

### A molecular basis for rBMP-2-induced ectopic ossification

**Fig. 4:**
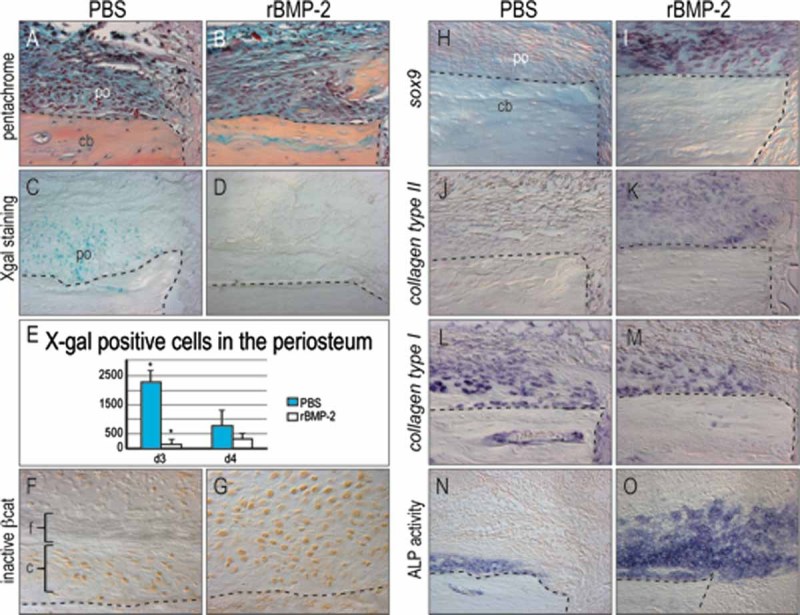
rBMP-2 induces differentiation of chondrocytes and upregulates osteochondroprogenitor markers in the extracortical space. On postoperative day 3, (*A*, *B*) pentachrome staining reveals a similar periosteal reaction in PBS- and rBMP-2-treated samples. (*C*) *Axin2^lacZ/+^* mice show an extensive distribution of β-catenin Wnt responsiveness in the injured periosteum. (*D*) rBMP-2 treatment abrogates this Wnt responsiveness here. (*E*) Quantification reveals a statistically significant reduction of Xgal^+^ cells on postoperative day 3. This difference is no longer present by day 4. (*F*) PBS-treated periosteum demonstrates minimal *sox9* expression of day 3. (*G*) rBMP-2-treated samples demonstrate a more robust and wider distribution of *sox9* expression in the periosteum. (*H*, *I*) Relative to PBS treatment, rBMP-2-treated samples exhibit higher *collagen type II* expression in the periosteum. (*J*, *K*) PBS- and rBMP-2-treated samples show similar *collagen type I* expression in the periosteum. (*L*, *M*) Relative to PBS-treated controls, rBMP-2-treated samples exhibit higher alkaline phosphatase activity in the periosteum. po = periosteum. *p < .01.

Simultaneous with its repression of osteogenesis in the bone marrow cavity, rBMP-2 treatment stimulated a robust chondrogenic response from periosteal cells (Fig. 1). Analyses on postoperative day 3 provided insights into how this response was elicited. The initial cellular response appeared equivalent between PBS- and rBMP-2-treated samples (Fig. 4*A, B*), but as it had in the bone marrow cavity, rBMP-2 repressed endogenous Wnt activity in the periosteum (Fig. 4*C, D*). Quantification of Xgal demonstrated that rBMP-2-treated samples had significantly less Wnt signaling activity than PBS-treated controls (*p* < .01; Fig. 4*E*). Staining for phosphorylated β-catenin demonstrated a preponderance of immunopositive cells in the rBMP-2-treated samples compared with the PBS-treated controls (Fig. 4*F, G*). Thus rBMP-2 inhibited endogenous Wnt signaling in the injured periosteum.

Wnt signaling represses *sox9*,(45) a transcriptional regulator of chondrogenesis.(46) Accordingly, in rBMP-2-treated samples, where endogenous Wnt signaling is repressed, we found stronger expression of *sox9* in the injured periosteum (Fig. 4*I*). PBS-treated controls, which exhibit robust Wnt signaling in the injured periosteum, showed very little *sox9* expression (Fig. 4*H*). *Sox9* directly regulates *collagen type II* transcription(47); as expected, rBMP-2-treated samples showed an upregulation in *collagen type II* expression relative to PBS-treated controls (Fig. 4*J, K*).

Both PBS- and rBMP-2-treated samples showed strong expression of *collagen type I* in the injured periosteum (Fig. 4*L, M*). In the rBMP-2-treated samples, the *collagen type I* domain overlapped with *collagen type II* (Fig. 4*K, M*), which indicates the commitment of skeletal progenitor cells to a chondrogenic lineage.(48,49) The domain of alkaline phosphatase activity also was expanded in rBMP-2-treated samples relative to PBS-treated controls (Fig. 4*N, O*). Thus rBMP-2 treatment inhibited endogenous Wnt signaling in the injured periosteum that coincided with an up regulation in *sox9* and *collagen type II* and a robust chondrogenic response.

**Fig. 5:**
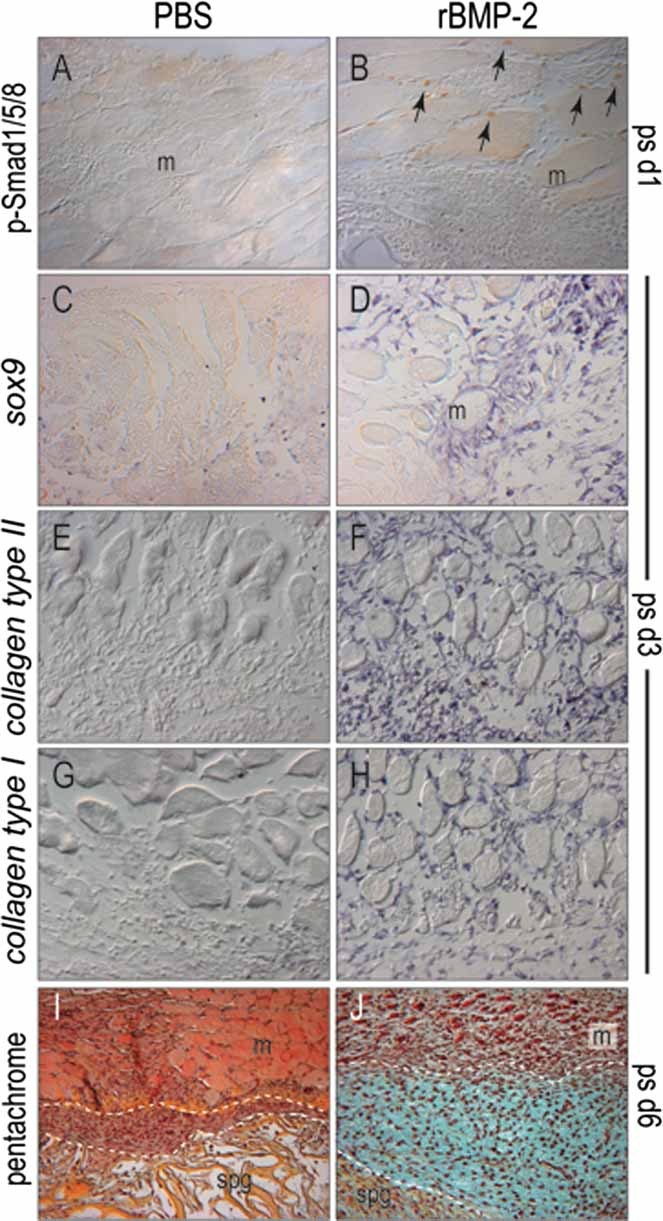
rBMP-2 induces osteochondroprogenitor markers in the supraperiosteal/muscle compartment. (*A*) PBS-treated muscle does not exhibit any Smad 1/5/8 phosphorylation on postoperative day 1. (*B*) rBMP-2 treatment induces phospho-Smad 1/5/8 immunostaining in this tissue by day 1 (*arrowheads*). (*C*, *D*) On postoperative day 3, pentachrome staining of both PBS- and rBMP-2-treated supraperiosteum reveals a highly cellular region with no overt osteogenesis or chondrogenesis in the supraperiosteal space. (*E*) PBS samples do not express *collagen type I*. (*F*) rBMP-2 treatment induces *collagen type I* expression. (*G*) PBS-treated samples do not express *collagen type II*. (*H*) rBMP-2 induces *collagen type II* expression here. (*I*) When the injury is modified to exclude the bone marrow cavity and separate the periosteal/supraperiosteal compartments, control samples exhibit only fibrous tissue between sponge and muscle (*outlined*). (*J*) rBMP-2-treated samples induce a separate chondrogenic response from supraperiosteal tissue in the defects. m = muscle; spg = sponge.

We also evaluated how cells in the supraperiosteal region responded to rBMP-2. Injury sites are closed by apposition of a muscle flap, which places this tissue in proximity to the sponge. On postoperative day 1, cells within the muscle flap responded to rBMP-2 as demonstrated by punctate phospho-Smad1/5/8 immunostaining; PBS-treated muscle flaps exhibited no immunopositive cells (Fig. 5*A, B*). In rBMP-2-treated samples, cells in the muscle flap had begun to differentiate into chondrocytes, as indicated by the coexpression of *sox9*, *collagen type II*, and *collagen type I* (Fig. 5*D, F, H*, respectively). These chondrogenic and osteogenic markers were not detectable in the muscle flaps of PBS-treated samples (Fig. 5*C, E, G*). Even using a modified injury that did not penetrate the cortex, supraperiosteal cells responded to the rBMP-2 stimulus by proliferating and undergoing chondrogenesis (Fig. 5*I, J*). Collectively, these data demonstrate that rBMP-2 treatment stimulated cells in the muscle flap to adopt a chondrogenic fate and contribute to the heterotopic callus that formed in the extracortical region.

## Discussion

Tissue regeneration increasingly is viewed as reactivation of developmental processes, but despite their similarities, there are significant differences as well. For example, postnatal skeletogenesis is influenced by the inflammatory response,(50) the mechanical environment,(51) innate differences among progenitor cell populations,(39,52) and a cell's state of differentiation at the time of exposure.(39) Thus it would be imprudent to assume that the regulatory functions of BMPs are equivalent across all stages and all locations of skeletogenic cell differentiation.

**Fig. 6:**
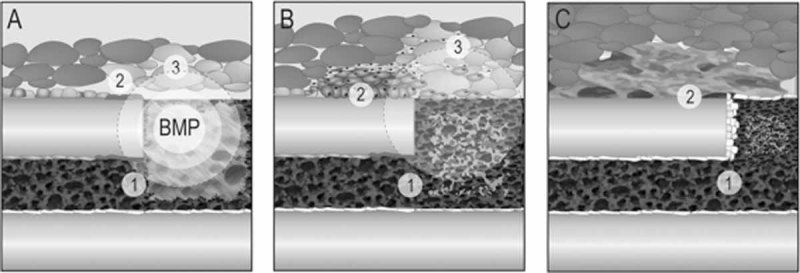
Tissue-specific responses to rBMP-2 can be used to predict the relative success of in vivo applications for the growth factor. (*A*) Following implantation, three populations of cells respond to rBMP: (1) cells in the bone marrow cavity, (2) cells in the injured periosteum, and (3) cells in the muscle overlying the injury site. Cells in the periosteum and endosteum are typically Wnt responsive (*blue cells*); rBMP treatment abrogates this responsiveness (*red cells*). (*B*) At an intermediate time point (in a mouse model, between 6 and 10 days after surgery), rBMP elicits three separate responses: (1) cells in the bone marrow subsequent to reduced Wnt signaling, neither proliferate nor differentiate into osteoblasts, (2) osteochondroprogenitor cells in the injured periosteum, which also exhibited a reduction in Wnt responsiveness at early time points, respond to rBMP by expressing *sox9* and adopting a chondrogenic fate, and (3) cells in the muscle respond to rBMP by adopting a chondrogenic fate, which contributes to the callus size. (*C*) At later stages of repair (in mice, around day 14), (1) the collagen sponge has resorbed, yet there is still no evidence of an osteogenic response from bone marrow cells, and (2) a coalescence of the periosteal and supraperiosteal cells creates a large extracortical bony bridge via the process of endochondral ossification.

With an objective towards translating insights from development into regenerative strategies, we examined the effects of rBMP on skeletal progenitor cells within a stereotypical injury site. Our data provide a mechanistic explanation for the apparently unpredictable responses observed in humans following use of rBMP. By employing an injury model in which skeletal stem/progenitor cell contributions could be distinguished from one another, we demonstrated that rBMP-2 has different effects depending on whether the progenitor cells originated from the periosteum, the endosteum and bone marrow cavity, or the surrounding musculature (Fig. 6).

### rBMP-2 inhibits osteoblast differentiation in the bone marrow cavity

There is a well-established feedback loop between BMP and Wnt signaling,(53,54) and Wnt signaling is a prerequisite for bone formation(55) and bone regeneration.(34,56) Accordingly, we tested whether rBMP-2 treatment blocked Wnt signaling and found that in both the injured bone marrow cavity and the injured periosteum, rBMP-2 treatment repressed endogenous Wnt signaling. This same feedback loop operates during fetal bone formation.(17) These data have clinical implications: There is accumulating evidence that activators of the Wnt pathway may be effective proosteogenic stimuli.(57) Consequently, the inhibitory effects of rBMP on Wnt signaling may be detrimental (i.e., in conditions where intramembranous ossification is preferred), but in other cases, where chondrogenesis is favored, it may be beneficial.

The inhibitory effect of rBMP-2 on bone marrow cells warrants further attention. Autologous bone marrow has inherent osteogenic potential,(58) and because of its limited availability,(59) investigators have searched for ways to augment or enhance its osteogenic capacity. In preclinical experiments, addition of rBMP-2 to bone marrow aspirate does not enhance osteogenesis. For example, rBMP-2 added to human bone marrow stromal cell cultures does not induce alkaline phosphatase activity and requires additional factors such as dexamethasone to induce osteoblast differentiation.(60–62) Likewise, the addition of rBMPs does not increase the osteogenic potential of grafted cancellous bone.(21,63)

Our data provide mechanistic insights into this antiosteogenic effect. In our model, rBMP-2-soaked sponges repressed osteogenesis in the endosteum and bone marrow cavity (Figs. 1 and 2). If left untreated, cells in the endosteum and bone marrow cavity normally differentiate into osteoblasts and generate new bone via intramembranous ossification (Figs. 1 and 2). rBMP-2 did not induce bone marrow cells to adopt a chondrogenic fate either, because *collagen type II* expression was undetectable (data not shown), and we did not find evidence of overt chondrogenesis at any of the time points examined. Moreover, rBMP-2 did not induce adipogenesis in the bone marrow cavity, as shown by oil red-O staining (data not shown). Instead, we found that rBMP-2 treatment repressed endogenous Wnt signaling (Fig. 3). Consequently, Wnt target genes such as *runx2*(64) are also repressed (Fig. 3). Runx2 is required for osteogenesis,(65) so the downregulation of *runx2* in the rBMP-2-treated bone marrow cavity is in keeping with the lack of bone formation in this site.

As mentioned earlier, the ability of rBMP-2 to inhibit osteogenesis via repression of Wnt pathway activity has a precedent in fetal skeletal development.(17) In this context, BMP signaling in osteoblasts limits bone mass through its action on sclerostin, which variously functions as an extracellular Wnt antagonist(66) and a BMP antagonist(67) (but see ref. (68)). We could not detect a reproducible effect of rBMP-2 treatment on *sclerostin* expression in the injury site, but given the pleiotropic functions of sclerostin/SOST, this may be a transient or subtle alteration in gene expression that is difficult to detect in an adult skeletal injury.

### rBMP-2 potently stimulates chondrogenic differentiation in the extracortical space

While rBMP-2 treatment inhibited osteogenesis in the bone marrow space, the protein had an entirely different effect on skeletal progenitor cells in the periosteum and extracortical region. Here, BMP exposure resulted in the upregulation of *collagen type II*, the stimulation of alkaline phosphatase activity, and a robust chondrogenic response (Fig. 5). The molecular mechanisms behind this robust chondrogenic response were revealed by analyses of *Axin2*^*LacZ/+*^ skeletal injuries. rBMP-2 downregulated Wnt activity in the periosteum (Fig. 4), which led to a derepression of Wnt target gene *sox9*.(45) In an embryonic context, Wnt signaling directly represses *sox9*,(45) which allows osteochondroprogenitor cells to adopt an osteogenic fate.(39)

Finally, by employing this injury model (Fig. 5), we gained insights into the mechanism responsible for BMP-mediated heterotopic ossification.(12) Specifically, rBMP-2 induces a robust chondrogenic response from cells in the periosteum and surrounding soft tissues (Fig. 5; and see refs. (69) to (71)), which leads to the upregulation of *sox9* and *collagen types I* and *II* and, ultimately, the differentiation of cells into a chondrogenic lineage (Fig. 5).

### Cell-dependent effects of rBMP-2

Within a single skeletal injury site, cell populations exhibit dramatically different responses to the same bone-inducing growth factor. In both the bone marrow cavity and the periosteum, rBMP-2 treatment represses the endogenous Wnt pathway, which is essential for intramembranous ossification in a healing skeletal defect.(34) How can the different responses be explained? Some new data suggest that while the periosteum contains osteochondroprogenitor cells, the endosteum only supports osteoprogenitor cells.(52) In embryonic osteochondroprogenitor cells, Wnt signaling is required for their differentiation into osteoblasts,(45) and when that Wnt signal is blocked, osteochondroprogenitor cells adopt a chondrogenic fate. In the endosteum, cells appear to have a more restricted potential; here, the lack of endogenous Wnt signaling causes them to arrest prior to differentiation into osteoblasts. In vitro, cells derived from the endosteum and bone marrow have the capacity to differentiate into chondrocytes.(72) This capacity is nonexistent (or repressed) in vivo, and therein may lie an explanation for the various effects attributed to rBMPs. Clearly, a better understanding of the in vivo response of skeletal progenitor cells will lead to improvements in the prochondrogenic and potentially proosteogenic effects of rBMP-2.
