# Supplementary material for: rBMP Represses Wnt Signaling and Influences Skeletal Progenitor Cell Fate Specification During Bone Repair
Source: J Bone Miner Res. 2010 Jan 15;25(6):1196–207. doi: 10.1002/jbmr.29 (PMC3153130; doi:10.1002/jbmr.29)
Supplement: Supplementary file 1 [file jbmr0025-1196-SD1.pdf]

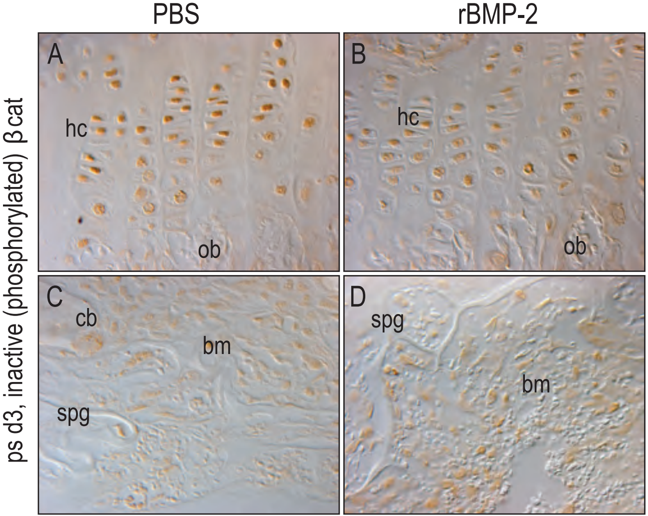

Supplemental Fig. 1
